# The Essential Role of Pin1 via NF-κB Signaling in Vascular Inflammation and Atherosclerosis in ApoE^−/−^ Mice

**DOI:** 10.3390/ijms18030644

**Published:** 2017-03-16

**Authors:** Ming Liu, Peng Yu, Hong Jiang, Xue Yang, Ji Zhao, Yunzeng Zou, Junbo Ge

**Affiliations:** Shanghai Institute of Cardiovascular Diseases, Institute of Clinical Science, Zhongshan Hospital, Shanghai Medical College, Fudan University, 180 Fenglin Road, Shanghai 200032, China; lmingw0559@163.com (M.L.); ypbaggio@163.com (P.Y.); sherrina_1010@126.com (X.Y.); zhao.ji@zs-hospital.sh.cn (J.Z.); zou.yunzeng@zs-hospital.sh.cn (Y.Z.); jbge@zs-hospital.sh.cn (J.G.)

**Keywords:** Pin1, NF-κB, inflammation, atherosclerosis

## Abstract

Atherosclerosis, as a chronic inflammatory disease, is the major underlying cause of death worldwide. However, the mechanisms that underlie the inflammatory process are not completely understood. Prolyl-isomerase-1 (Pin1), as a unique peptidyl-prolyl isomerase, plays an important role in inflammation and endothelial dysfunction. Herein, we investigate whether Pin1 regulates vascular inflammation and atherosclerosis, and clarify its mechanisms in these processes. ApoE^−/−^ mice were randomly given either juglone (0.3, 1 mg/kg, two times per week) or vehicle i.p. for 4 weeks. Compared with ApoE^−/−^ mice, treatment by juglone resulted not only in markedly attenuated macrophage infiltration and atherosclerotic lesion area in a lipid-independent manner, but also in decreased expression of Pin1, vascular cell adhesion molecule-1 (VCAM-1), monocyte chemoattractant protein-1 (MCP-1), and NF-κB activity in aorta. Then, EA.hy926 cells were pretreated with juglone (6 μmol/L), Pin1 siRNA, NF-κB inhibitor, or vehicle prior to exposure to ox-LDL (50 μg/mL). It was observed that treatment with juglone or Pin1 siRNA suppressed expression of VCAM-1 in oxLDL-incubated EA.hy926 cells and decreased THP-1 cell adhesion to oxLDL-stimulated endothelial cells through the NF-κB signal pathway. Our findings indicate that Pin1 plays a vital role on the development of vascular inflammation and atherosclerosis.

## 1. Introduction

Atherosclerosis, as a chronic inflammatory disease, is the major underlying cause of death worldwide. In the early stage, endothelial dysfunction is a critical event in atherosclerosis, which recruits macrophages into intima and contributes to the initiation of atherosclerosis [1,2]. Hence, elucidating the molecular mechanisms that underlie the inflammatory process in early atherosclerosis conduces to the development of novel intervention strategies for atherosclerosis.

Prolyl-isomerase-1 (Pin1), as a unique peptidyl-prolyl isomerase, is an enzyme that regulates cellular functions through conformational changes of proteins [3]. By isomerizing specific phosphorylated Ser/Thr-Pro bonds, Pin1 alters the structures and activities in certain proteins, including protein kinases, protein phosphatases, transcription activators, and regulators [3,4]. It has been reported that Pin1 plays a pivotal role in chronic inflammation diseases such as rheumatoid arthritis [5], asthma [6], and liver fibrosis [7]. Recent data revealed that Pin1-dependent conformational changes reduced nitric oxide availability and induced endothelial dysfunction [8]. Moreover, Pin1 inhibition prevents diabetes-induced endothelial dysfunction via NF-κB signaling [9]. Nonetheless, the pathophysiological role of Pin1 in atherosclerosis remains unknown. Herein, we investigated whether Pin1 regulates vascular inflammation and atherosclerosis, and clarified its mechanism in these processes.

## 2. Results

### 2.1. Inhibition of Pin1 Attenuates Atherosclerosis in ApoE^−/−^ Mice

**Figure 1:**
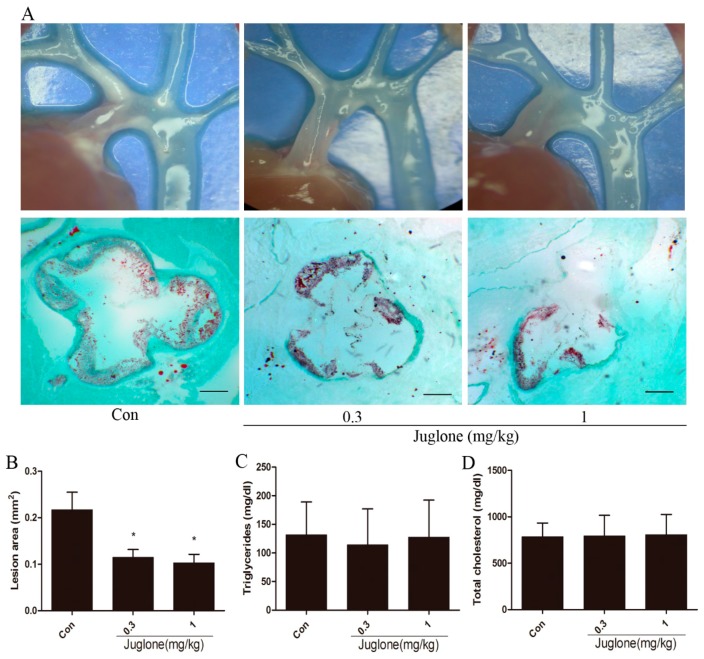
Pin1 impacts atherosclerosis in ApoE^−/−^ mice. After four weeks of respective treatment, 18-week-old ApoE^−/−^ mice were sacrificed. (**A**) Shown were representative pictures of aortic arches (20×) and aortic sinus sections stained with oil red O (Scale bar = 50 μm); (**B**) Atherosclerotic lesion area in the aortic sinus was quantified (*n* = 8). The serum triglyceride (**C**) and total cholesterol (**D**) levels were measured (*n* = 6). * *p* < 0.05 vs. the control group.

To study the functional role of Pin1 in atherogenesis, we treated ApoE^−/−^ mice with a Pin1 inhibitor. After 4 weeks of respective intervention, aortic sinus assays were performed to evaluate lesion formation. Compared with the control group, the atherosclerotic lesion area was markedly reduced in juglone-treated ApoE^−/−^ mice (≈47% reduction in the 0.3 mg/kg group; ≈53% reduction in 1 mg/kg group) (Figure 1A,B).

In addition, serum lipid levels were determined. As shown in Figure 1C,D, there were no differences in serum total cholesterol or triglyceride levels in these three groups of mice. Together, these results suggest that inhibition of Pin1 attenuates atherosclerosis by a lipid-independent manner.

### 2.2. Inhibition of Pin1 Suppresses Vascular Inflammation in ApoE^−/−^ Mice

**Figure 2:**
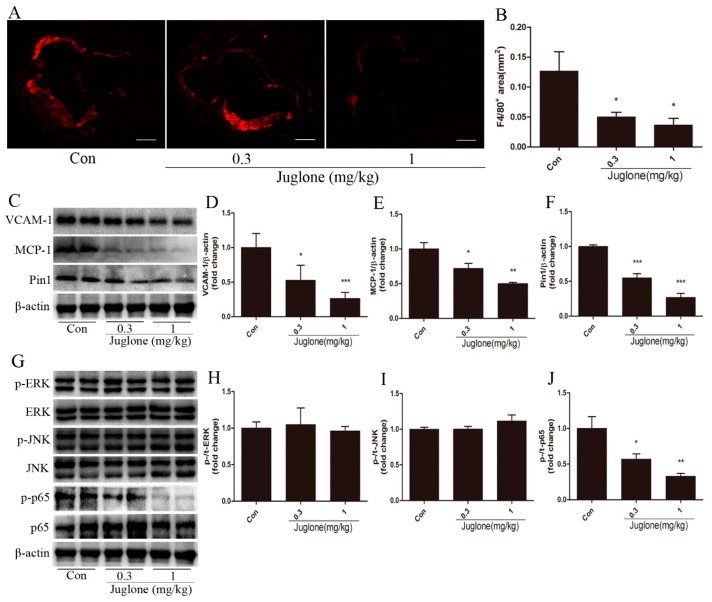
Pin1 impacts vascular inflammation and signaling pathways in the aortas of ApoE^−/−^ mice. At ages of 18 weeks, ApoE^−/−^ mice were sacrificed, and aortas were retrieved. The levels of macrophages accumulation in atherosclerotic lesions were examined by immunohistochemical staining of F4/80 (**A**) and quantified (**B**) (*n* = 7, scale bar = 50 μm); (**C**–**F**) Aortic tissues from indicated mice were subjected to Western blot for the detection of VCAM-1 (**D**), MCP-1 (**E**), and Pin1 (**F**) expression, normalized by levels of β-actin (*n* = 4), and the representative bands are shown (**C**); (**G**–**J**) Signal transduction in aortas was examined by Western blot: (**H**) for ERK, (**I**) for JNK, and (**J**) for NF-κB (*n* = 4), and the representative bands are shown (**G**). * *p* < 0.05, ** *p* < 0.01, *** *p* < 0.001 vs. the control group.

To investigate the effect of Pin1 on vascular inflammation, we determined the levels of macrophages infiltration. After 4 weeks of treatment, F4/80^+^ macrophage content in atherosclerotic lesions was significantly reduced in juglone-treated ApoE^−/−^ mice (≈60% reduction in 0.3 mg/kg group; ≈71% reduction in 1 mg/kg group) compared with the control group (Figure 2A,B).

Subsequently, we further determined two inflammatory molecules, VCAM-1 and MCP-1, which contributed to macrophage migration and atherosclerosis [1,10]. In line with the decreases in the lesion area and macrophage infiltration, expressions of VCAM-1 and MCP-1 were markedly reduced in ApoE^−/−^ mice treated with juglone versus a vehicle (Figure 2C–E). In addition, we proved the inhibitory effect of juglone on Pin1. Compared with the control group, the expression of Pin1 in the aorta was significantly reduced in ApoE^−/−^ mice treated with juglone (Figure 2C,F). Taken together, these findings indicate that Pin1 may play a critical role in the progression of vascular inflammation and atherosclerosis.

### 2.3. NF-κB Activation is Regulated by Pin1 in ApoE^−/−^ Mice

To clarify whether the ERK, JNK, and NF-κB signal pathways are involved in the Pin1-dependent regulation of vascular inflammation and atherosclerosis, we determined the activities of ERK, JNK, and NF-κB in the aortas. In comparison with the control group, the protein level of phosphorylated (active) p65 was lower in juglone-treated ApoE^−/−^ mice (Figure 2G,J). However, the phosphorylation of ERK or JNK was similar between the three groups of mice (Figure 2G–I). The data suggest that the NF-κB signaling pathway may be involved in Pin1-mediated vascular inflammation and atherosclerosis.

### 2.4. Pin1 Regulates oxLDL-Induced Inflammatory Response in EA.hy926 Cells

**Figure 3:**
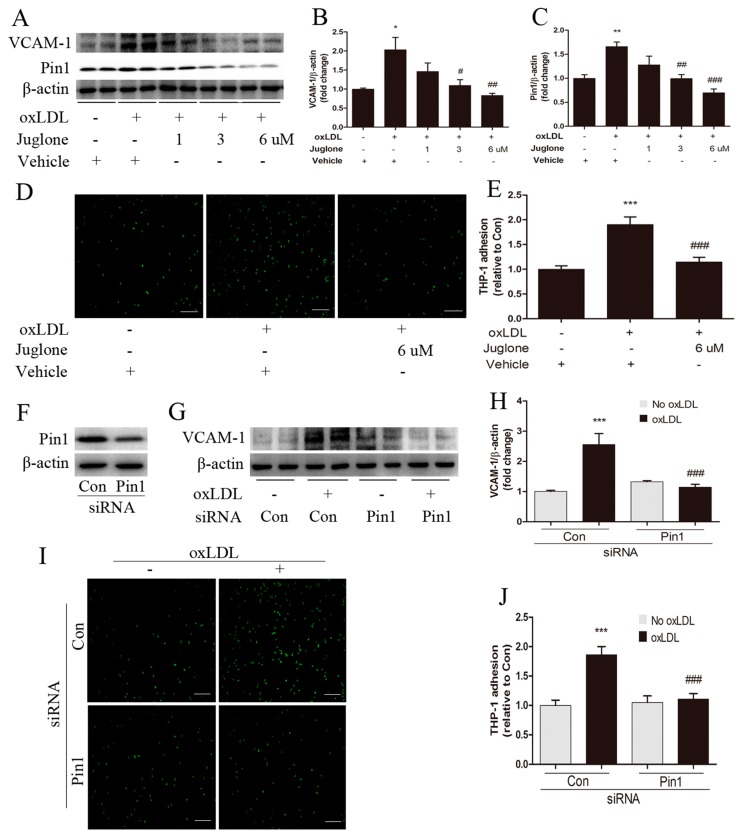
Pin1 impacts inflammatory response in oxLDL-induced EA.hy926 cells. Endothelial cells were pretreated with a vehicle or juglone (1, 3, 6 μM) for 1 h prior to incubation with oxLDL (50 μg/mL) for 24 h, as indicated. (**A**–**C**) Western blot was performed for the detection of VCAM-1 (**B**) and Pin1 (**C**) expression, normalized by levels of β-actin (*n* = 4), and the representative bands are shown (**A**); (**D**,**E**) THP1 cells (in green) adhesion to EA.hy926 cells treated with juglone was measured as described in METHODS (*n* = 6–8, Scale bar = 50 μm); EA.hy926 cells were stimulated with oxLDL (50 μg/mL) for 24 h after transfection with the control (Con) or Pin1 siRNA. Western blot was performed for the detection of Pin1 (**F**) and VCAM-1 (**G**,**H**) expression, normalized by levels of β-actin (*n* = 4); (**I**,**J**) Adhesion of THP1 cells to EA.hy926 cells transfected with siRNA to knock down Pin1 (*n* = 6, Scale bar = 50 μm). * *p* < 0.05, ** *p* < 0.01, *** *p* < 0.001 vs. the vehicle group or the Con group; # *p* < 0.05, ## *p* < 0.01, ### *p* < 0.001 vs. the oxLDL-induced group.

To investigate whether Pin1 might modulate the inflammatory response in endothelial cells, we assessed the effects of Pin1 on the expression of VCAM-1 in EA.hy926 cells and the level of monocyte-endothelial cell adhesion. Monocyte adhesion assay revealed an increased THP-1 cell adhesion to oxLDL-stimulated EA.hy926 cells, which had a higher VCAM-1 and Pin1 expression (Figure 3A–E). Interestingly, inhibition of Pin1 by juglone decreased VCAM-1 and Pin1 expression in a dose-dependent effect, and reduced THP-1 cell adhesion to oxLDL-stimulated EA.hy926 cells (Figure 3A–E), which is in line with our in vivo results. Furthermore, siRNA-mediated Pin1 knockdown in EA.hy926 cells also decreased oxLDL-induced VCAM-1 expression and attenuated THP-1 cell adhesion to oxLDL-stimulated EA.hy926 cells (Figure 3F–J). Thereby, we confirm the critical role of Pin1 in oxLDL-induced proinflammatory endothelial phenotype.

### 2.5. NF-κB Signaling Involves in Pin1-Dependent Regulation of Inflammatory Response in EA.hy926 Cells

**Figure 4:**
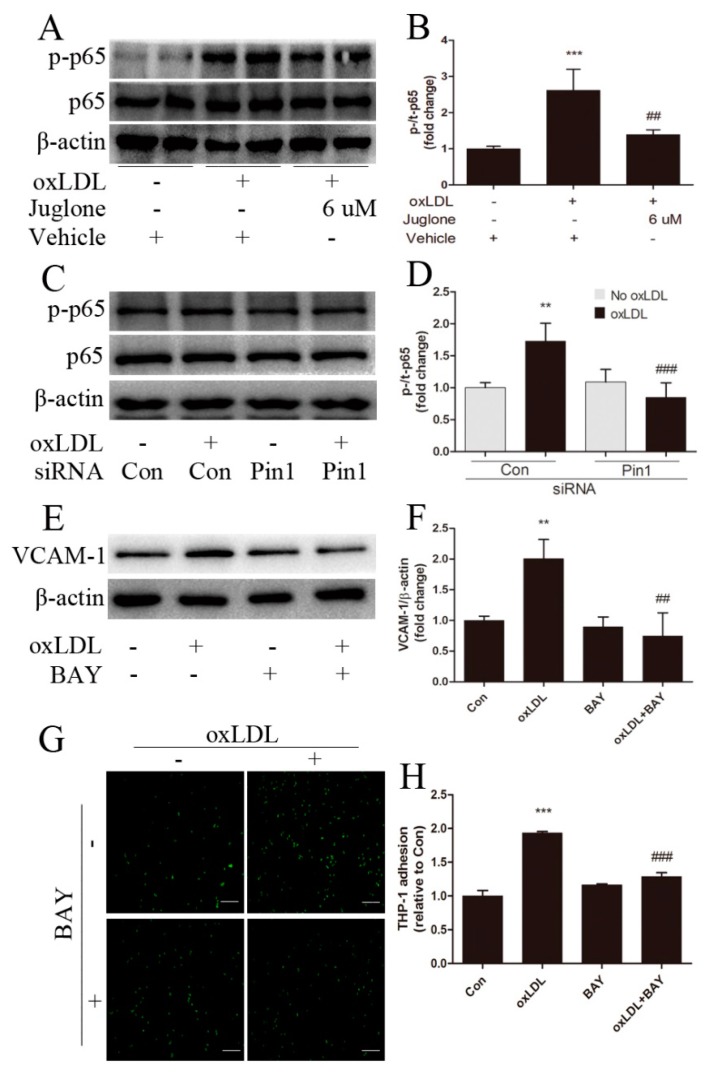
The role of NF-κB signaling in Pin1-regulated inflammatory response induced by oxLDL in EA.hy926 cells. NF-κB signal transduction in endothelial cells stimulated with oxLDL (50 μg/mL) for 15 min after pretreatment with the vehicle or juglone (6 μM) for 1 h (**A**,**B**) or transfection with the control (Con) or Pin1 siRNA (**C**,**D**) (*n* = 4); EA.hy926 cells were pretreated with the vehicle or BAY11-7082 (5 μM) for 1 h prior to incubation with oxLDL (50 μg/mL) for 24 h, as indicated; (**E**,**F**) Western blot was performed for detection of VCAM-1 expression, normalized by levels of β-actin (*n* = 4); (**G**,**H**) Adhesion of THP1 cells to EA.hy926 cells pretreated with BAY11-7082 (BAY) to inhibit the NF-κB signal pathway (*n* = 6, Scale bar = 50 μm). ** *p* < 0.01, *** *p* < 0.001 vs. the vehicle group or the Con group; ## *p* < 0.01, ### *p* < 0.001 vs. the oxLDL-induced group.

To further elucidate the signal transduction pathways mediating the impact of Pin1 on regulation of oxLDL-induced inflammatory response in EA.hy926 cells, we tested the effect of NF-κB signaling. Incubation of EA.hy926 cells with oxLDL induced a significant increase in phospho-p65, compared with the vehicle group (Figure 4A,B). Consistent with our in vivo observations, a marked reduction in p-p65 was found in oxLDL-stimulated EA.hy926 cells in the presence of Pin1 inhibition by juglone or Pin1 siRNA compared with that observed in the presence of the vehicle or the control (Figure 4A–D).

We then determined whether NF-κB signaling accounts for oxLDL-induced inflammatory response in EA.hy926 cells. The endothelial cells were pretreated with an inhibitor of NF-κB. Compared with oxLDL stimulation, pretreatment with NF-κB inhibitor BAY11-7082 resulted in a highly significant reduction in oxLDL-induced upregulation of VCAM-1 and attenuated THP-1 cell adhesion to oxLDL-stimulated EA.hy926 cells (Figure 4E–H). These observations suggested that Pin1 reduced inflammatory response in oxLDL-incubated EA.hy926 cells, in part via NF-κB signaling.

## 3. Discussion

Atherosclerosis, as a chronic inflammatory disease, is initiated by endothelial dysfunction. It has been reported that Pin1 plays a pivotal role in chronic inflammation diseases such as rheumatoid arthritis [5], asthma [6], and liver fibrosis [7]. Meanwhile, evidence suggests that Pin1-dependent conformational changes reduce nitric oxide availability and induce endothelial dysfunction [8]. Moreover, Pin1 inhibition prevents diabetes-induced endothelial dysfunction [9]. However, it is unknown whether Pin1 is involved in the development of atherosclerosis.

In the present study, we uncovered three important insights into roles for Pin1 in atherosclerosis. First, Pin1 inhibition suppressed vascular inflammation and atherosclerosis as well as activation of NF-κB signaling in ApoE^−/−^ mice; Second, Pin1 inhibition improved oxLDL-induced inflammatory response in endothelial cells; Third, Pin1 inhibition suppressed the activation of NF-κB and decreased endothelial cells activation induced by oxLDL, at least partly via NF-κB signaling.

Endothelial dysfunction and upregulation of VCAM-1 and MCP-1 are critical events in atherosclerosis, which recruits macrophages into intima and contributes to the initiation of atherosclerosis [1,2,11]. It has been reported that Pin1 reduces nitric oxide availability and subsequently induces endothelial dysfunction [8]. Moreover, Pin1 inhibition prevents diabetes-induced expressions of VCAM-1 and MCP-1 and endothelial dysfunction [9]. Consistent with these findings, our data also support that Pin1 plays a critical role in vascular inflammation and atherosclerosis. We found that a Pin1 inhibitor significantly decreased the expressions of VCAM-1 and MCP-1 in the aorta of ApoE^−/−^ mice. Pin1 inhibition by the inhibitor also prevented macrophage infiltration into lesions. Moreover, Pin1 inhibition by either inhibitor treatment or gene knockdown decreased the VCAM-1 protein level induced by oxLDL and attenuated THP-1 cell adhesion to oxLDL-stimulated EA.hy926 cells. In line with reduced vascular inflammation by the Pin1 inhibitor, the decrease in oil red O staining was observed in Pin1 inhibitor-treated ApoE^−/−^ mice. However, we did not find a significant difference in the lipid profiles between ApoE^−/−^ mice treated with the vehicle and those treated with the Pin1 inhibitor. Therefore, these findings suggest that Pin1 inhibition may improve vascular inflammation and consequently suppress atherosclerotic progression beyond lipid lowering.

As a unique peptidyl-prolyl isomerase, Pin1 isomerizes specific phosphorylated Ser/Thr-Pro bonds in certain proteins, including protein kinases, protein phosphatases, transcription activators, and regulators [3,4]. Such Pin1-mediated conformational changes regulate the structures and activities of these proteins [12]. It has been reported that Pin1 can regulate the activities of ERK, JNK, and NF-κB [3,9,12,13]. Meanwhile, these signal pathways play important roles in endothelial dysfunction [2,9,14,15]. However, it is yet unknown which is involved in Pin1-regulated vascular inflammation and atherosclerosis in ApoE^−/−^ mice. The results of this study revealed that Pin1 inhibition helped to decrease the activation of the NF-κB signal pathway in the aortas of ApoE^−/−^ mice, but not ERK or JNK signal pathways. Consistent with in vivo findings, Pin1 inhibition by either inhibitor treatment or gene knockdown also reduced activation of NF-κB signaling induced by oxLDL in EA.hy926 cells, not ERK or JNK signal pathways [16]. Moreover, pretreatment of the NF-κB inhibitor decreased the VCAM-1 expression induced by oxLDL and attenuated THP-1 cell adhesion to oxLDL-stimulated EA.hy926 cells. Similar to our results, Costantino et al. [9] revealed that Pin1 inhibition prevented diabetes-induced VCAM-1 expression and endothelial dysfunction via NF-κB signaling. Taken together, these results indicate that the NF-κB signal pathway is involved in Pin1-regulated vascular inflammation and atherosclerosis.

In summary, our findings confirmed the critical role of Pin1 in the early stage of vascular inflammation and atherosclerosis. However, the role of Pin1 in vulnerable plaque is still poorly understood. Undoubtedly, further studies are needed to clarify the underlying mechanisms of Pin1 in unstable plaque.

## 4. Materials and Methods

### 4.1. Reagents and Antibodies

Dulbecco’s Modified Eagle Medium (DMEM) and Roswell Park Memorial Institute (RPMI) 1640 were purchased from KeyGEN BioTECH (Nanjing, China). Fetal bovine serum (FBS) was obtained from GIBCO (New York, NY, USA). Juglone (a inhibitor of Pin1), DMSO, and oil red O were purchased from Sigma-Aldrich (St. Louis, MO, USA). BAY11-7082 and oxLDL were obtained from Meilunbio (Dalian, China). Calcein-AM was purchased from YEASEN (Shanghai, China). The primary antibodies against VCAM-1 and F4/80 were obtained from Abcam (Cambridge, UK). Rabbit antibodies against MCP-1, ERK1/2, p-ERK1/2, JNK, p-JNK, p65, and p-p65 were purchased from Cell Signaling Technology (Beverly, MA, USA). Rabbit anti-Pin1 antibody was obtained from Proteintech (Chicago, IL, USA). HRP-conjugated antibody against β-actin was purchased from Kangchen Biotechnology (Shanghai, China).

### 4.2. Mice

Animal studies were performed in accordance with the guidelines in the Institutional Animal Care and Use Committee of Shanghai Model Organisms Center, Inc., Shanghai, China (2015-0018, 25 December 2015). Twenty-four male ApoE^−/−^ mice (5 weeks old) were provided by Shanghai Model Organisms Center, Inc. (Shanghai, China) and housed in microisolator cages under specific pathogen-free conditions at the Shanghai Model Organisms Center, Inc. All mice were fed with a Western diet (21% fat, 0.15% cholesterol) for 13 weeks. At 14 weeks of age, ApoE^−/−^ mice were randomized to treatment with either juglone (0.3, 1 mg/kg, two times per week) or a vehicle (0.3% DMSO) i.p. for 4 weeks. All mice were sacrificed at 18 weeks old.

### 4.3. Quantification of Atherosclerotic Lesions

Mice were fasted for over 4 h and then anesthetized. Aortic arches were dissected, and photographs were taken. Next, the aortic roots were harvested and immediately fixed in ice-cold 4% paraformaldehyde (PFA) for 4 h. After washing in PBS for 15 min 3 times, fixed aortic roots were dehydrated in 30% sucrose overnight. Then, they were embedded at the optimal cutting temperature (OCT) compound (Sakura, Torranc, CA, USA), and snap-frozen. The aortic sinuses were cut into 10 μm serial sections for further analysis. The atherosclerotic lesion were stained with oil red O and quantified by ImageJ software 1.41o (National Institutes of Health, Bethesda, MD, USA), as previously described [11].

### 4.4. Cell Culture and Treatment

EA.hy926 cells, a human endothelial cell line, were purchased from Cell Bank of Chinese Academy of Sciences (Shanghai, China) and cultured in DMEM supplemented with 10% fetal bovine serum and antibiotics at 37 °C in 5% CO_2_ and 95% room air. For cellular assays, we switched the cells to DMEM containing 1% FBS. Next, 80% confluent cells were stimulated with the indicated dose and time of juglone, BAY11-7082 or vehicle, prior to incubation with oxLDL (50 μg/mL) for 15 min or 24 h. Likewise, we transfected the cells with 100 nM of siRNA (all from Genomeditech, Shanghai, China) targeting Pin1 or with scrambled siRNA using the riboFECT™ CP regent (RiboBio Co., Ltd., Guangzhou, China). The cells were stimulated with oxLDL (50 μg/mL) 48 h after siRNA transfection.

### 4.5. Monocyte-Endothelial Cell Adhesion Assay

EA.hy926 cells were grown to confluence in 24-well plates and treated with oxLDL ± juglone, Pin1 siRNA or BAY11-7082. Next, THP1-1 cells were purchased from Cell Bank of Chinese Academy of Sciences (Shanghai, China) and grown in RPMI 1640 supplemented with 10% fetal bovine serum and antibiotics at 37 °C in 5% CO_2_ and 95% room air. After being washed twice with PBS, THP-1 cells were resuspended in serum-free RPMI 1640 with 3 μM Calcein-AM and incubated for 30 min at 37 °C. EA.hy926 cells were washed twice with DMEM and incubated with 400 μL of serum-free DMEM. Then, 10^5^ cells/100 μL of labeled THP-1 cells were overlaid on EA.hy926 cells and incubated for 1 h at 37 °C. After incubation, the unbound THP-1 cells were removed by washing with PBS and the attached THP-1 cells on the endothelial cells monolayer were fixed with 4% PFA for 10 min. Ten pictures per well were taken at a 100× magnification, and the number of attached THP-1 cells per field of view was analyzed by ImageJ software, as previously described [17].

### 4.6. Western Blot Analysis

Aortic tissues and EA.hy926 cells were retrieved and subjected to disruption by an ultrasonic homogenizer in RIPA containing protease and phosphatase inhibitors (Pierce Biotechnology, Rockford, IL, USA). Protein concentrations were determined using an assay from Pierce (Rockford, IL, USA). Equal amounts of protein were separated on 10% SDS-PAGE gels; Western blotting was performed as previously described [11]. The primary antibodies included VCAM-1(1:800), MCP-1 (1:800), Pin1 (1:1000), ERK1/2 (1:1000), p-ERK1/2 (1:1000), JNK (1:1000), p-JNK (1:1000), p65 (1:1000), p-p65 (Ser536; 1:1000), and β-actin (1:5000). Signals were visualized using the enhanced chemiluminescence (ECL) detection system (Pierce Biotechnology, Rockford, IL, USA). Quantitative analysis of the band density was performed using Quantity One software 4.6.2 (Bio-Rad, Berkeley, CA, USA). All bands were normalized to β-actin.

### 4.7. Immunohistochemistry

For immunostaining, PFA-fixed cryostat sections were rehydrated in PBS and blocked for 30 min in PBS containing 5% BSA, 2% nonimmune serum solution at room temperature. Sections were probed with rat anti-F4/80 antibody (1:150 dilution) in blocking buffer overnight at 4 °C. After incubation, sections were washed three times with PBS and incubated with goat anti-rat IgG conjugated with Alexa Flour 555 (1:200; Invitrogen, Carlsbad, CA, USA) diluted in blocking buffer at room temperature for 2 h. Quantitative analysis of the F4/80 positive areas in the lesions used an ImageJ software.

### 4.8. Serum Lipid Analysis

Fasted mice were anesthetized, and blood was drawn from the right ventricle. Levels of total cholesterol and triglyceride in serum were measured by colorimetric enzymatic assays (Jinan Sysmex Limited Company, Jinan, China).

### 4.9. Statistical Analysis

Data were expressed as mean ± SEM. All statistical analyses were performed using GraphPad Prism software 5.0 (GraphPad Software, Inc., La Jolla, CA, USA). Multiple group comparisons were performed by one-way ANOVA followed by the post-hoc with Tukey test. *p* < 0.05 was considered statistically significant.

## 5. Conclusions

In summary, our findings demonstrate that Pin1 inhibition suppresses vascular inflammation and atherosclerosis as well as the activation of NF-κB signaling in ApoE^−/−^ mice. Moreover, inhibition of Pin1 improves oxLDL-induced inflammatory response in endothelial cells, at least partly via NF-κB signaling. Taken together, our results implicate Pin1 in mechanisms underlying endothelial dysfunction and vascular inflammation in atherosclerosis. Given the essential role of endothelial dysfunction in the initiation of atherosclerosis, Pin1 downregulation or inhibition may be a candidate for preventing atherosclerosis.

## Abbreviations

Pin1	prolyl-isomerase-1
ApoE^−/−^	apolipoprotein E deficient
VCAM-1	vascular cell adhesion molecule-1
MCP-1	monocyte chemoattractant protein-1
siRNA	small interfering RNA
OxLDL	oxidative low density lipoproteins
NF-κB	nuclear factor kappa-B
ERK	extracellular regulated protein kinases
JNK	c-Jun N-terminal kinase
THP1	human acute monocytic leukemia cell line
